# Cost of delivering childhood RSV prevention interventions to the health system in Kenya: a prospective analysis

**DOI:** 10.1136/bmjopen-2024-084207

**Published:** 2024-11-21

**Authors:** Ranju Baral, Elkanah Otiang, Joseph Odiyo, Bryan O Nyawanda, Joyce U Nyiro, Patrick Munywoki, Rose E Jalang'O, Clint Pecenka

**Affiliations:** 1 Center for Vaccine Innovation and Access, PATH, Seattle, Washington, USA; 2 PATH, Nairobi, Kenya; 3 Independent Consultant, Nairobi, Kenya; 4 Kenya Medical Research Institute, Center for Global Health Research, Kisumu, Kenya; 5 Kenya Medical Research Institute–Wellcome Trust Research Programme, Kilifi, Kenya; 6 National Vaccines and Immunization Program, Ministry of Health, Nairobi, Kenya

**Keywords:** health economics, respiratory infections, infectious diseases

## Abstract

**Abstract:**

**Objectives:**

To evaluate the cost of delivering childhood respiratory syncytial virus (RSV) prevention interventions to the health system in Kenya.

**Design:**

A prospective (cost projection) activity-based costing study.

**Setting:**

Kenya, national introduction of interventions.

**Participants:**

Not applicable.

**Interventions:**

A single-dose RSV maternal vaccine and a single-dose, long-acting monoclonal antibody (mAb).

**Primary and secondary outcome measures:**

Cost per eligible target population; cost per dose administered; non-commodity cost of delivery. Costs are reported in 2023 USD.

**Results:**

RSV interventions are expected to be delivered using existing systems: maternal vaccine using the antenatal care platform and the mAb delivered similar to existing birth dose vaccines. Assuming a price of US$3 per dose (for both interventions) and baseline coverage rates averaging 50% for the maternal vaccine and 86% for the mAb, the estimated cost of delivering maternal vaccine was US$1.74 (financial) and US$6.60 (economic) per vaccinated woman, and the cost of delivering mAbs was US$1.56 (financial) and US$6.27 (economic) per vaccinated child. Excluding commodity cost, the cost of delivering maternal vaccine was US$1.32 (financial) and US$2.72 (economic) and that for mAb was US$1.23 (financial) and US$2.48 (economic). Cost differences between the two interventions are driven by the anticipated baseline coverage. Health worker training, service delivery and programme planning and coordination were major cost drivers.

**Conclusion:**

This study presents the prospective cost of new RSV intervention introduction and delivery in low-income and middle-income country settings, which is largely unknown. Cost estimates incorporate anticipated health system strengthening activities needed to deliver the future RSV interventions. These cost estimates support country-level and global-level decision-makers evaluating implementation feasibility and intervention affordability.

**Strengths and limitations of this study**

- This is one of the first studies examining potential respiratory syncytial virus prevention intervention delivery costs in low-income and middle-income countries.
- In-country experts and programme representatives advised and validated key model inputs and assumptions.
- This costing study builds on the current country capacity reflecting the country-specific resource needs at the time of analysis.

## Introduction

 Respiratory syncytial virus (RSV) is a common cause of acute lower respiratory illness among children, contributing to more than 100 000 deaths among children under 5 years of age globally.^1^ RSV infection is most severe in young infants and the burden is disproportionately high among children in low-income and middle-income countries (LMICs), where more than 97% of RSV deaths occur.^1^ Given the severity of RSV among younger children and especially young infants, they are one of the primary target groups for disease prevention strategies.

Advancements in RSV interventions that target disease prevention in young infants^2 3^ have shown promising results. Recently, a long-acting monoclonal antibody (mAb) (Nirsevimab, AstraZeneca) and a maternal vaccine (Abrysvo, Pfizer) were approved by the US Food and Drug Administration.^4 5^ Global stakeholders are optimistic that these RSV prevention products—once available and recommended for broader use—can be successfully integrated into the existing health system platforms across LMICs, where they are needed most. Gavi, the Vaccine Alliance (Gavi), is committed to supporting these interventions for LMICs,^6^ contingent on the availability of a product that is licensed, WHO prequalified and available at a reasonable price. The delivery strategies for these new RSV interventions, however, are likely going to be context specific and may require adaptations to the existing immunisation delivery system.

As global and country decision-makers explore feasible mechanisms to deliver new RSV interventions, cost of delivery is one critical factor for consideration. With this study, we sought to generate empirical evidence on the potential costs of delivering a single-dose RSV maternal vaccine and a single-dose long-acting RSV mAbs within the health system in Kenya. Kenya is one of the few LMICs with a well-established respiratory surveillance system and has identified a notable RSV disease burden among children.^7^,^10^ Recent estimates suggest an annual incidence of outpatient and non-medically attended RSV associated acute respiratory infections in Kenya at 206 and 226 per 1000 children under 5 years of age, respectively.^7^ This awareness of disease is an important factor for discussing potential intervention options and associated delivery scenarios.

While evidence on costs of delivering childhood immunisation programmes across LMICs is growing, cost of delivery evidence for maternal vaccines is nearly non-existent.^11 12^ This study aims to fill this gap by estimating RSV intervention introduction and delivery costs to the Kenyan health system. Understanding the costs associated with delivering future RSV interventions will allow both in-country decision-makers and global policymakers to better understand the cost implications of the different intervention and delivery strategies and will inform decision-making and planning around further use of the interventions. The cost of delivery estimates generated from this study can also be used to inform cost-effectiveness and to help understand the economic feasibility of implementing such interventions as they become available.

## Methods

### Scenarios for programme implementation and costing

Both mAbs and maternal vaccines are relatively new to most immunisation programmes across LMICs. RSV mAbs are designed to be administered directly to the newborn, similar to the birth dose BCG vaccination, for example. RSV maternal vaccines are designed to be administered to pregnant women during a specified gestation window, allowing timely antibody transfer to protect the newborn from RSV infection. A number of vaccines are currently given safely and effectively during pregnancy, such as tetanus toxoid-containing vaccines and influenza and pertussis vaccines. Most of the existing maternal vaccines administered in LMICs leverage parts of the antenatal care (ANC) delivery platform. However, given the novelty of RSV interventions, existing immunisation and ANC delivery systems would need adaptations to successfully introduce and deliver these products, which has potential cost consequences.

We held a 1-day stakeholder meeting in Kenya in June 2022 to discuss feasible delivery strategies for successfully implementing RSV prevention programmes within the Kenyan health system. Forty-four participants, including the national and select county Ministry of Health (MOH) representatives, academics, researchers and representatives from various non-governmental organisations, contributed to the deliberation.^13^ In Kenya, most women receive vaccines at Expanded Programme on Immunisation (EPI) clinics, following referral from the ANC visits. Children (including newborns) are vaccinated at EPI clinics. The convening concluded that upcoming RSV interventions are best delivered by leveraging the existing service delivery mechanisms in Kenya and that existing maternal tetanus vaccines and birth dose BCG vaccines provide proven pathways for implementing both new interventions. The convening also identified several key areas where the health system needs to be strengthened prior to the availability of RSV interventions. Following the recommendations of the convening, this study generated cost estimates for the following scenarios:

1. A single-dose, long-acting mAb delivered to newborns, using existing immunisation visits timed closely after birth; and
2. A single-dose maternal vaccine delivered to pregnant women during ANC contact.

### Scope and perspective

Neither maternal RSV vaccine nor RSV mAb are currently available or in use in Kenya. We thus took a prospective (cost projection) approach to project costs, from the health systems perspective, of introducing and delivering these interventions in Kenya. The health systems perspective was chosen because vaccination is primarily the function of the Kenya MOH. Cost projections cover a 5-year period beginning in 2023 and assume national introduction across all counties. All pregnant women and all newborns in Kenya during the study period are assumed eligible to receive a single dose of RSV maternal vaccine and a long-acting RSV mAb, respectively. Activities identified as necessary for both interventions are considered financial costs to the government, irrespective of the financing mechanisms. At the time of new vaccine introduction, however, countries may be supported by external donor agencies or through grants from Gavi. The value of the existing resource use, including the opportunity cost of existing staff time and donated goods, is included in the economic costs.

### Costing approach

We used an ingredients-based activity costing approach to generate cost projections. We first identified and individually costed all potential activities associated with the introduction and delivery of RSV interventions at all levels of health system, including making necessary adaptations to the existing system. Activities were then grouped into standard categories associated with vaccination programmes, such as programme planning and management, procurement and distribution, training, etc (see online supplemental table 1). We mapped resource requirements for each activity using the quantity of resources and their unit costs to generate cost projections.

The costing analysis followed the standard guidelines for estimating the potential costs of new vaccine introduction and delivery^14^,^17^ and only included the incremental cost of introducing and delivering the interventions into the existing or adapted delivery platforms. The incremental cost analysis considered all potential additional costs that are not already being paid for by the existing health system.

### Data

Following the stakeholder consultation, the study team worked closely with the immunisation and maternal health programmes responsible for providing both immunisation and ANC in Kenya to develop detailed activity maps that identified the subactivities necessary to implement each new intervention. We determined these activities through costing interviews with EPI and ANC programme leads and focal points, in which they discussed recent experiences with new vaccine introductions and programme expectations. Specific calls for any system-wide changes and adaptations of the existing healthcare delivery system that would be needed to create conducive platforms for RSV interventions were also discussed and incorporated into the costing. Detailed activities included in the costing analysis are in online supplemental table 1.

We collected data from representative samples of health administrative units, vaccine stores and health facilities to inform the potential recurring costs at regional, subregional and facility levels, with inputs from the MOH. We selected five counties across Kenya (Kilifi, Kirinyaga, Marsabit, Nakuru and Siaya) to capture geographic variations. We then selected three health facilities in each county (n=15) from which to collect data on recurrent activities, including service delivery. We collected data from both antenatal service delivery units and immunisation delivery units at the sample health facilities. Additionally, we collected data from the national vaccine store (n=1), regional vaccine stores (n=2) and subcounty vaccine stores (n=5) to estimate the cost of cold chain management, storage and vaccine distribution. We also discussed additional activities (at all levels) necessary for RSV intervention introduction and delivery and added them to the detailed activity map developed at the national level. A list of counties, subcounties and health facilities selected for data collection is included in online supplemental table 2. All data collection occurred between November 2022 and March 2023.

The vaccine quantities and supplies used for cost projections were derived based on the projected target population (pregnant women and birth cohort for maternal vaccine and mAb, respectively). The target population was adjusted for the anticipated coverage provided by the national immunisation programme. Immunisation programme representatives in Kenya validated the consolidated data on all subactivities and the assumptions on respective input resources and unit costs used to generate the final cost estimates. Cost data were collected in local currency units (LCU) and are reported in both LCU and USD 2023 units.

### Shared inputs and capacity considerations

RSV interventions under consideration in this study are currently unavailable for the LMIC market. We therefore assumed both products would be delivered as a single-dose regimen (based on the target product profile^18^ and their use in the clinical trial setting^19^). We assumed both vaccine and mAb doses would be donated to the country, so product price was considered only in economic cost estimates. The cost of procurement add-on (such as shipping and handling, clearance) on both products were assumed to be paid by the government, thus included in financial cost estimates. In Kenya, typically, UNICEF procures and delivers vaccines and mAbs and supplies to the national vaccine store (central vaccine store) based on agreed shipment schedule (usually monthly). In the absence of data on actual procurement add-on costs, which can largely depend on the volume of shipment and the product characteristics, among other factors, we made assumptions based on the anticipated product characteristics and the volume requirements for both maternal vaccines and mAbs.

For either product, the cold chain storage volume requirements were assumed based on the anticipated product characteristics (table 1) and anticipated requirement for quantity of doses. Costs of additional cold chain capacity requirements were calculated based on assumed cold chain volume requirements for storage at each level of the health facility. To account for the incremental resource requirements for distribution in a routine setting, inputs shared with the existing system are attributed to RSV products based on direct allocation (10%), informed by our study survey.

**Table 1:** Key data inputs and assumptions used in the analysis

Inputs	RSV maternal vaccine	RSV mAb	Data source
**Target population and coverage**			
Target group	Pregnant women	Newborns	TPP of products
Target population in 2022	1 668 242	1 616 060	MOH Kenya
Population growth rate	3%	3%	MOH Kenya
Coverage rate, average across counties	50% (range: 16% to 74%)	86% (range: 69%–100%)	MOH Kenya- Kenya Health Information System
**Product characteristics**			
Presentation	Three doses per 2 mL vial, lyophilised	Two dose vial, liquid	Personal note from Bill & Melinda Gates Foundation (Gates Foundation)
Diluent	Three doses per 2 mL vial	N/A	Personal note from Gates Foundation
Vaccine packaged volume (cm^3^/dose)	9.3	9.3	Assumed, based on RTS,S vaccine
Reconstitution syringe per dose administration	0.33	N/A	Assumed, personal note from Gates Foundation
Doses per course	Single	Single	Assumed, based on TPP and clinical trial design
**Cost of commodities**			
Cost of vaccine per dose	US$3	US$3	Assumed
Cost per injection syringe	US$0.05	US$0.05	Assumed, based on Kenya MVIP
Cost per reconstitution syringe	US$0.20	N/A	Assumed, based on Kenya MVIP
Cost per safety box (100 syringe capacity)	US$1	US$1	Assumed, based on Kenya MVIP
Procurement add-on charges as a % of product cost	7.60%	7.60%	Assumed, based on Kenya MVIP
Freight, insurance, inspection	4.60%	4.60%	
Handling fees	3%	3%	
**Service delivery**			
Proportion of vaccination in routine in-facility setting	80%	80%	Assumed
Proportion of vaccination in outreach setting	20%	20%	Assumed
Average time to vaccinate one person, routine in facility setting	8.6 min (range: 3 min–15 min)	8.8 min (range: 5 min–15 min)	Health facility survey
Average time to vaccinate one person, outreach setting	7.5 min (range: 5 min–10 min)	8.3 min (range: 3 min–15 min)	Health facility survey
**Others**			
Cold chain capacity expansion requirements	National level: Purchase 1 cold room (10 m^3^) in the introduction year. Regional level: Purchase 1 cold room (10 m^3^) in the introduction year. Subcounty level: Purchase 2 cold boxes per subcounty in the introduction year. Health facility level: Purchase 1 cold box per health facility in the introduction year.		Approximation, informed by health facility survey
Useful life years for capital investments	10 years		Assumed
Useful life of introduction activities	5 years		Assumed
Discount rate	3%		Assumed
Exchange rate (1 USD =)	134 Kenyan shilling (KSH)		Oanda, April 2023^25^

mAbmonoclonal antibodyMOHMinistry of HealthMVIPMalaria Vaccine Implementation ProgrammeRSVrespiratory syncytial virusTPPtarget product profile

To account for the opportunity cost of staff time spent on all RSV introduction and delivery activities, we used the Kenya MOH salary scale by health worker cadre type for year 2020 based on salaries from the Salaries and Renumeration Commissions, Kenya. Time required to complete each activity was then multiplied by the average salary within a given cadre to approximate opportunity time costs. The incremental time required per maternal vaccine or mAb administration was based on average vaccination time during tetanus-diphtheria vaccine administration (for maternal vaccine) and routine EPI vaccination (for mAb), both derived from health facility surveys and reported by health workers. For other needed resources, we assumed 100% existing spare capacity in the immunisation system to accommodate RSV interventions.

### Cost estimates

For both RSV interventions, cost estimates are presented as the incremental cost per dose administered and cost of delivery (non-commodity cost) per dose. The single-dose schedule assumed for both products means the cost per full vaccination is equivalent to cost per dose administered. The annualised cost of introduction/initial set-up costs for the study duration were added to the recurrent costs across all activity categories during the study period to generate the total cost of the programme. The cost per dose administered is calculated by dividing the total cost of the programme by the total number of doses administered throughout the duration of the analysis. The cost of delivery per dose is calculated by subtracting the commodity cost (vaccine and immunisation supplies) and the procurement add-on costs from the total cost and dividing by the total number of doses delivered.

Data were aggregated across the sample health facilities and administrative units and the average values were used in the cost projections.

### Sensitivity analysis

For both RSV interventions, baseline cost estimates were generated using the input values and assumptions noted in table 1. We conducted a one-way sensitivity analysis to estimate the sensitivity of cost estimates with different input values for a subset of variables—for example, vaccine price, coverage, wastage rate and service delivery time. We used observed minimum and maximum values, identified from health facility surveys, to generate a range of cost estimates. Results with alternative input values are presented separately and in tornado graphs.

### Patient public involvement

No patients were involved in this study. We have included roles and relationships between different members of the research team in the reflexivity statement (see online supplemental file).

This study was approved by the Kenyatta National Hospital-University of Nairobi (KNH-UoN ERC) Ethics Review Committee (approval number P802/10/2021).

## Results

### Target population, projected vaccinations

The number of expected pregnant women in Kenya in 2022 was 1 668 242. Using an average annual population growth rate of 3%, the projected total target population for maternal vaccine during the study period (2023–2027) was 9 122 631. Similarly, the total number of live births in 2022 was 1 616 060 and the projected total target population for mAbs during the study period was 8 837 278 (table 2). We used observed ANC 4+ coverage by county for the year 2022^20^ as the projected coverage rate for maternal vaccine. Similarly, the observed BCG vaccine coverage rate for year 2022^20^ was used as the coverage rate for mAb. The coverage was constant over the study duration. Over a 5-year study period, roughly 4 696 192 pregnant women and/or 7 720 980 infants were expected to be vaccinated.

**Table 2:** Target population, projected vaccinations and total cost (in USD), 2023–2027

Metric	RSV maternal vaccine		RSV mAb	
	All years	Annual average	All years	Annual average
Target population				
Total target population	9 122 631	1 824 526	8 837 278	1 767 456
Total number of doses delivered	4 696 192	939 238	7 720 980	1 544 196
Total cost				
Total cost, financial	12 341 929	2 468 386	13 876 761	2 775 352
Total cost, economic	36 397 153	7 279 431	49 822 749	9 964 550
Total cost, introduction cost, financial	7 530 869	1 506 174	7 530 869	1 506 174
Total cost, introduction cost, economic	11 136 161	2 227 232	11 136 161	2 227 232
Total cost, recurrent cost, financial	4 811 059	962 212	6 345 892	1 269 178
Total cost, recurrent cost, economic	25 260 993	5 052 199	38 686 588	7 737 318

mAbmonoclonal antibodyRSVrespiratory syncytial virus

### Cost of RSV interventions delivery

Under baseline input assumptions (table 1), RSV maternal vaccine introduction costs are estimated to be US$1.25 (financial) and US$3.99 (economic) per eligible target population. Total costs including annualised introduction and annual recurrent cost are US$1.74 (financial) and US$6.60 (economic) per vaccinated pregnant women. The cost of delivery, excluding commodity costs, is estimated at US$1.32 (financial) and $2.72 (economic) per vaccinated pregnant women.

For RSV mAbs, the introduction costs are estimated to be US$1.46 (financial) and US$5.64 (economic) per eligible target population. Total costs including annualised introduction and annual recurrent cost is US$1.56 (financial) and US$6.27 (economic) per vaccinated child. The cost of delivery, excluding commodity costs, is estimated at US$1.23 (financial) and US$2.48 (economic) per vaccinated child. The total incremental financial and economic cost of both RSV interventions are provided in table 3.

**Table 3:** Unit cost estimates of RSV interventions delivery in Kenya (in USD)

Metric	RSV maternal vaccine		RSV mAb	
	Financial	Economic	Financial	Economic
Cost per eligible target population	1.25	3.99	1.46	5.64
Introduction cost per eligible target population	0.72	1.22	0.74	1.26
Recurrent cost per dose administered	1.02	5.38	0.82	5.01
Cost per dose administered	1.74	6.60	1.56	6.27
Cost of delivery per dose, excluding commodity	1.32	2.72	1.23	2.48

mAbmonoclonal antibodyRSVrespiratory syncytial virus

### Cost drivers and sensitivity

The initial set-up or introduction cost constitutes 58% (financial) and 31% (economic) of the total cost for maternal vaccine. For mAb, the initial set-up or introduction cost constitutes 51% (financial) and 22% (economic) of the total costs.

Training constitutes the major cost driver for about 30% of the financial costs for both maternal vaccine and mAb. Similarly, training accounts for 16% and 12% of economic costs for maternal vaccine and mAb, respectively. Besides training, procurement constitutes the major cost drivers for both interventions accounting for roughly 20% of the total financial cost and more than 50% of total economic cost. Service delivery accounted for about 12% and 18% of total financial cost for maternal vaccine and mAb, respectively.

On recurrent costs (financial), the main cost drivers for maternal vaccine were procurement (41%), service delivery (29%), distribution and storage (11%) and supervision (9%). The cost drivers of the recurrent cost (financial) for mAb were procurement (41%), service delivery (36%), distribution and storage (8%) and supervision (7%). For both interventions, procurement costs were the single major cost driver of economic recurrent cost accounting for more than 70% of cost (see online supplemental table 3).

**Figure 1:**
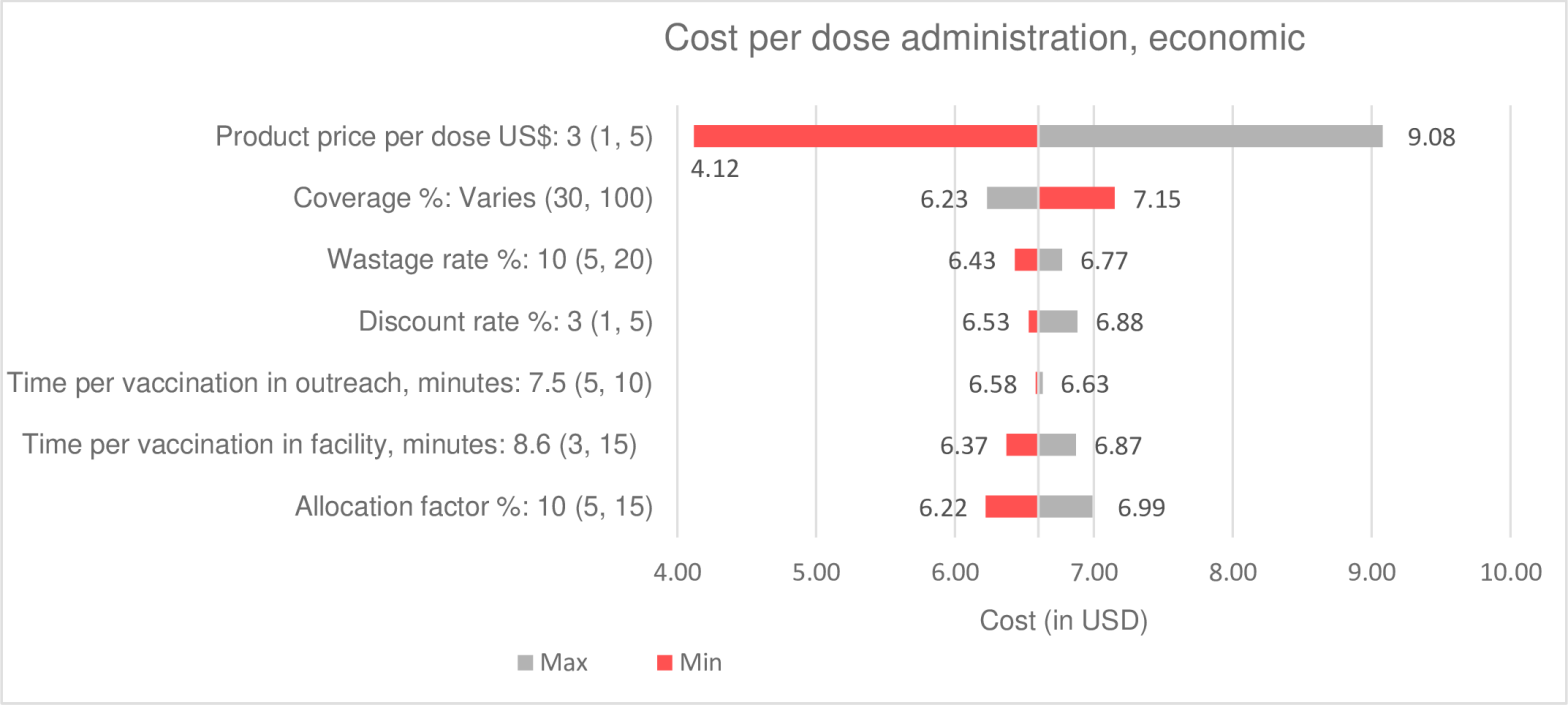
One-way sensitivity of unit cost estimates for RSV maternal vaccine introduction and delivery. RSV, respiratory syncytial virus.

Results of the one-way sensitivity analysis of cost estimates with different input values are presented in a tornado graph (see figures1  2). The cost per dose administered estimates for both maternal vaccine and mAb are most sensitive to product price, coverage and the allocation factor.

**Figure 2:**
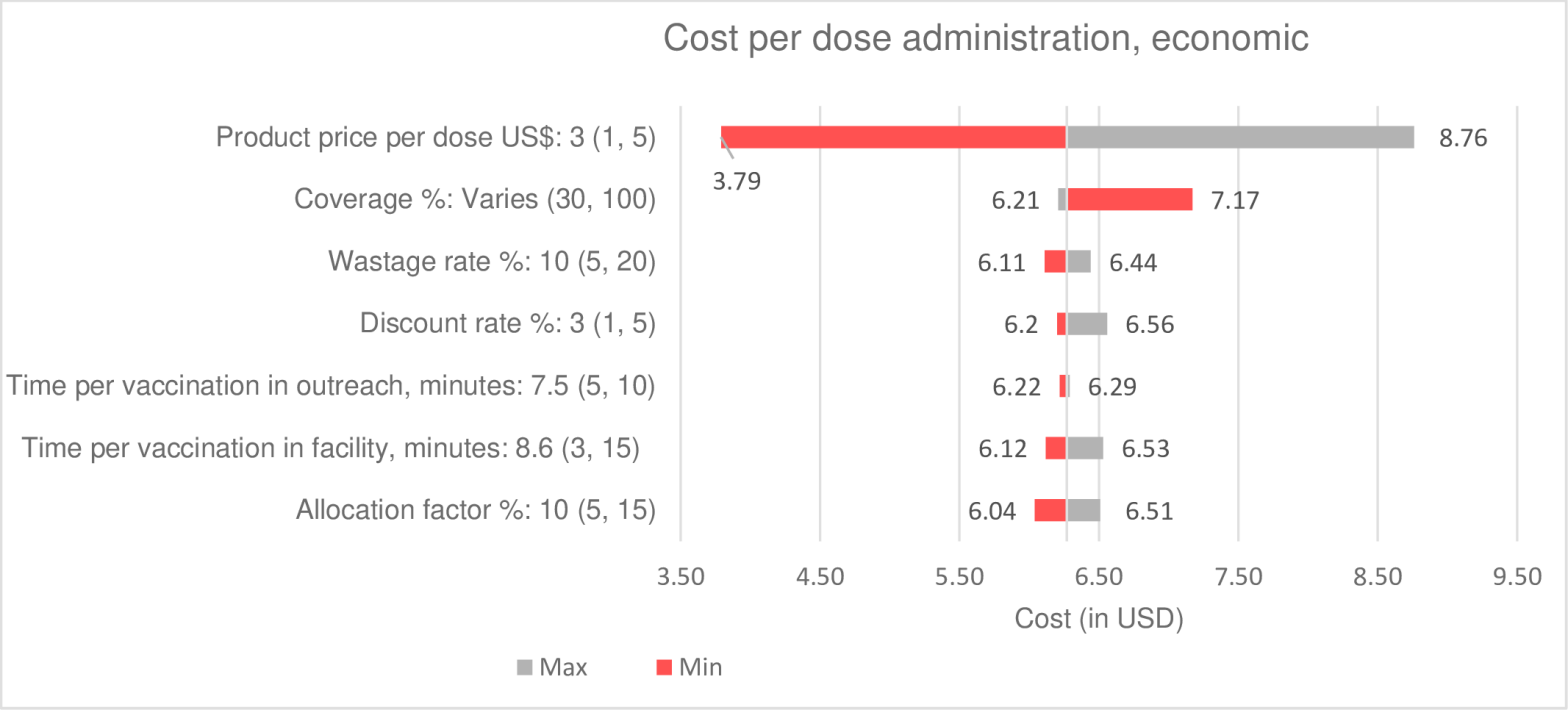
One-way sensitivity of unit cost estimates for RSV mAb introduction and delivery. mAb, monoclonal antibody; RSV, respiratory syncytial virus.

## Discussion

Vaccine products that target pregnant women and mAbs that target newborns represent new intervention approaches, and their implementation will likely require adaptations to existing service delivery approaches.^21^ These adaptations will have cost consequences for national immunisation programmes. While the literature on the cost of new vaccine implementation and delivery across LMICs is growing,^11 12^ particularly for childhood vaccination, to our knowledge there are few data on the cost of delivering maternal vaccine or mAb in LMIC settings. This study helps stakeholders understand what it could cost to introduce and deliver RSV maternal vaccine and mAb via the existing healthcare delivery system in LMICs, specifically Kenya. Findings from this study are informative to both country-level and global-level decision-makers who are evaluating the feasibility and affordability of implementing such interventions.

The cost estimates generated in this paper incorporate anticipated health system strengthening activities needed to deliver the forthcoming RSV maternal vaccine and birth dose mAbs. The cost analysis is built on insights from the in-country stakeholder’s workshop held in Kenya,^13^ where immunisation and maternal health programme leaders came together to discuss the best delivery approaches for RSV maternal vaccine and mAbs and the adaptations necessary for their successful implementation. Strengthening the existing tetanus-diphtheria delivery mechanisms (where pregnant women are vaccinated during routine ANC visits) was deemed the most efficient approach to maternal vaccine delivery in Kenya; for mAb, the existing newborn vaccine delivery system was identified as the most feasible approach.

Our findings suggest that, in Kenya, the unit cost of introducing and delivering new maternal RSV vaccine is slightly higher (US$1.74 per vaccinated women) than introducing RSV mAb (US$1.56 per vaccinated child). The financial unit cost of introduction activities is not that different for the two RSV interventions (US$0.72 vs US$0.74). This reflects the similar types of activities with comparable intensity needed to introduce both interventions, as identified by country stakeholders. Further, in Kenya, the frontline health workforce provides both immunisation and ANC services and has similar basic training with transferable skillsets. ANC and EPI staff rotate periodically, providing both cadre of service providers with experience supporting both pregnant women and children.

A relatively large eligible target population for maternal vaccine drives down the unit cost per eligible women compared with that for mAb. However, the recurrent costs of delivery (per vaccinated person) are slightly higher for maternal vaccine (US$1.02) compared with mAb (US$0.82). The anticipated higher coverage for mAb (based on observed BCG coverage rates in Kenya) lowers the unit cost per dose administered by almost 20 cents per dose (financial) when compared with maternal vaccine cost per dose administered (based on observed ANC coverage rates in Kenya). Higher maternal vaccine coverage would result in similar delivery cost estimates across both interventions. Further, if the introduction costs are spread over a longer timeframe, it would lower the total cost per unit of delivery for both interventions.

Training is the single major cost driver, accounting for more than 50% of the introduction/initial set-up costs for both interventions. Knowledge of RSV disease is relatively low among health workers.^22^ Increasing awareness among health workers at all levels via comprehensive training would be an important component of programme introduction, but it would contribute to higher training costs for both interventions. Also, supervision of health workers, especially during the year new products are first introduced, would contribute to higher costs for this activity. Targeted communication and social mobilisation strategies would be critical for ensuring vaccine demand and attaining coverage for both and substantially contribute to the cost of introducing both interventions.

Several recent costing studies evaluated the cost of delivering malaria vaccine to children through the routine immunisation programme in Kenya.^23 24^ No studies on the costs of delivering maternal immunisation interventions in Kenya exist. A recent review paper found only a handful of studies generating cost of delivery estimates for maternal immunisation and only 2 of the 16 studies included in the review were from LMICs. Based on the review’s findings, the cost per dose of delivering maternal immunisation alongside an existing ANC service provision range from US$0.55 to US$0.64 in low-income counties, US$1.25 to US$6.55 in middle-income countries, and US$5.76 to US$39.87 in high-income countries.^12^ The cost of introducing and delivering the forthcoming RSV interventions are within the comparable range of other new vaccine introduction costs in Kenya.^23 24^

One of the strengths of this paper is that the cost analysis is built on input from in-country stakeholders. Detailed activities were derived following a series of discussions with the implementers of both the maternal health and immunisation programmes. Deliberations were followed by a review of previous new vaccine introductions and identification of new activities that would facilitate the anticipated system adaptations necessary for introduction. These discussions were strengthened with data from health facility surveys across the country. The cost estimates we generated are realistic as they address country needs for successful implementation of such programmes.

One of the limitations of this study is that the activities costed reflect the existing health system capacities and resources needed to fill the local capacity gaps, which may limit the generalisability of the findings in different settings. Findings from this prospective costing study nonetheless generate useful insights to help assess economic feasibility and affordability of the new products in the Kenyan context. Interpreting cost projections nonetheless should be done carefully as it builds on informed assumptions rather than actual expenditure data. Lack of data on the product cost and product characteristics extends to the higher uncertainty in cost estimates, especially for total procurement costs, and the cold chain capacity strengthening needs. Given neither product is yet available for the LMIC market, we assumed similar prices for both products which may be overly simplistic. Nevertheless, the cost exclusive of commodity costs (ie, delivery costs) are useful for countries assessing the long-term recurrent costs associated with new vaccine product introduction and delivery. Reasonable assumptions used in the baseline analysis and the sensitivity analysis provide a range of cost estimates and can serve as a starting point for discussions.

This analysis does not consider the availability of financing to support forthcoming RSV interventions. Ultimately, product introduction decisions, product choice and programme sustainability will depend on the availability of financing (Gavi) and other external support available to the country. Affordability of new intervention products, especially in the face of economic challenges and transition from Gavi support, mean that countries will need to evaluate the interventions in the context of broader health priorities and needs.

## supplementary material

10.1136/bmjopen-2024-084207 online supplemental file 1

## Data Availability

All data relevant to the study are included in the article or uploaded as supplementary information.

## References

[1] Li Y, Wang X, Blau DM (2022). Global, regional, and national disease burden estimates of acute lower respiratory infections due to respiratory syncytial virus in children younger than 5 years in 2019: a systematic analysis. Lancet.

[2] Higgins D, Trujillo C, Keech C (2016). Advances in RSV vaccine research and development - A global agenda. Vaccine (Auckl).

[3] PATH (2023). RSV vaccine and mab snapshot (PDF). https://www.path.org/resources/rsv-vaccine-and-mab-snapshot/.

[4] U.S. Food and Drug Administration (2023). FDA approves new drug to prevent RSV in babies and toddlers. https://www.fda.gov/news-events/press-announcements/fda-approves-new-drug-prevent-rsv-babies-and-toddlers?mkt_tok=NDkwLUVIWi05OTkAAAGNCE1ROEGQBpF-yxHvsQZW0bt59F-4tiISkjNrR82lSLUZAYDupXUoEF9M2PcvFpcYwZv4ByowAhhob1Y0FYLNg6mU12pyNhZVg1N_h-evRvbl.

[5] Pfizer (2023). FDA advisory committee votes in support of approval for pfizer’s vaccine candidate to help prevent RSV in infants through maternal immunization. https://www.pfizer.com/news/press-release/press-release-detail/fda-advisory-committee-votes-support-approval-pfizers.

[6] Gavi (2018). Vaccine investment strategy 2018. https://www.gavi.org/our-alliance/strategy/vaccine-investment-strategy-2018.

[7] Nyawanda BO, Murunga N, Otieno NA (2023). Estimates of the national burden of respiratory syncytial virus in Kenyan children aged under 5 years, 2010-2018. BMC Med.

[8] Nokes DJ, Ngama M, Bett A (2009). Incidence and severity of respiratory syncytial virus pneumonia in rural Kenyan children identified through hospital surveillance. Clin Infect Dis.

[9] Bigogo GM, Breiman RF, Feikin DR (2013). Epidemiology of respiratory syncytial virus infection in rural and urban Kenya. J Infect Dis.

[10] Berkley JA, Munywoki P, Ngama M (2010). Viral etiology of severe pneumonia among Kenyan infants and children. JAMA.

[11] Immunizationeconomics.org (2023). Immunization delivery cost catalogue. https://immunizationeconomics.org/thinkwell-idcc.

[12] Procter SR, Salman O, Pecenka C (2020). A review of the costs of delivering maternal immunisation during pregnancy. Vaccine (Auckl).

[13] PATH & KEMRI Wellcome Trust (2022). Childhood rsv burden and prevention strategies in kenya: June 2022 RSV stakeholders convening.

[14] Logan B (2014). Common approach for the costing and financing of routine immunization and new vaccines (EPIC). https://static1.squarespace.com/static/556deb8ee4b08a534b8360e7/t/62bb16c83c114a5786d72900/1656428265663/FINAL+EPIC+Common+Approach+Working+Paper+0814.pdf.

[15] World Health Organization (2002). Guidelines for estimating costs of introducing new vaccines into the national immunization system. https://iris.who.int/handle/10665/67342.

[16] World Health Organization-Immunization, Vaccines and Biologicals (2002). Guidelines for estimating costs of introducing new vaccines into the national immunization system. https://iris.who.int/bitstream/handle/10665/329389/WHO-IVB-19.10-eng.pdf.

[17] Levin A, Boonstoppel L, Brenzel L (2022). WHO-led consensus statement on vaccine delivery costing: process, methods, and findings. BMC Med.

[18] World Health Organization (2017). WHO preferred product characteristics for respiratory syncytial virus (RSV) vaccines. https://iris.who.int/bitstream/handle/10665/258705/WHO-IVB-17.11-eng.pdf?sequence=1.

[19] World Health Organization (2021). WHO preferred product characteristics of monoclonal antibodies for passive immunization against respiratory syncytial virus (‎RSV)‎. https://iris.who.int/bitstream/handle/10665/341635/9789240021853-eng.pdf?sequence=1.

[20] Ministry of Health (2023). Kenya health information system [dataset]. https://hiskenya.org/dhis-web-commons/security/login.action.

[21] Krishnaswamy S, Lambach P, Giles ML (2019). Key considerations for successful implementation of maternal immunization programs in low and middle income countries. Hum Vaccin Immunother.

[22] Nyawanda BO, Opere VA, Nyiro JU (2023). Respiratory Syncytial Virus (RSV) Disease and Prevention Products: Knowledge, Attitudes, and Preferences of Kenyan Healthcare Workers in Two Counties in 2021. Vaccines (Basel).

[23] Baral R, Levin A, Odero C (2021). Costs of continuing RTS,S/ASO1E malaria vaccination in the three malaria vaccine pilot implementation countries. PLoS One.

[24] Baral R, Levin A, Odero C (2023). Cost of introducing and delivering RTS,S/AS01 malaria vaccine within the malaria vaccine implementation program. Vaccine (Auckl).

[25] Oanda (2024). Oanda currency converter. https://www.oanda.com/currency-converter/en/?from=USD&to=KES&amount=1.

