# Supplementary material for: Cost of delivering childhood RSV prevention interventions to the health system in Kenya: a prospective analysis
Source: BMJ Open. 2024 Nov 21;14(11):e084207. doi: 10.1136/bmjopen-2024-084207 (PMC11590859; doi:10.1136/bmjopen-2024-084207)
Supplement: online supplemental file 1 [file bmjopen-14-11-s001.pdf]

Supplementary tables

Appendix Table 1: Detailed activities included in the costing analysis.

Activities	Frequency of activities
**Vaccine and injection supplies procurement**	
Procurement of vaccine doses and mAb	Each year
Procurement of supplies- reconstitution syringe	Each year for maternal vaccine. None for mAb
Procurement of supplies- administration syringe	Each year
Procurement of supplies- Safety boxes	Each year
**Distribution**	
Receive vaccine and supplies at the national store	Monthly delivered by contracted third party, shared cost
Distribution of vaccine and supplies from national store to regional stores	Quarterly (4 times) a year, shared cost
Collection of vaccine and supplies from regional store by sub-county stores	Quarterly (4 times) a year, shared cost
Collection of vaccine and supplies from sub-counties by health facilities	Monthly collection, shared cost
Operating cost of cold chain and maintenance at National, regional, and health facility levels	Monthly, shared cost
**Program planning and coordination**	
Hold Kenya Immunization Technical Advisory Group (KENITAG) meetings for introduction decisions	Once per quarter in the introduction year only
KENITAG sub-committee meetings	Once per month in the introduction year only
Vaccine Implementation steering committee meeting to develop plans for introduction at the National level	Once in the introduction year
County level microplanning for vaccine introduction	Once in the introduction year
Sub-county level microplanning for vaccine introduction	Once in the introduction year
Routine microplanning at national, county and sub-county levels	Each year, shared cost
**Training**	
Training sub-committee meetings to develop training plans	Once a month in the introduction year
Workshop to develop training packages, tools adaptation, and printing of training tools	Once in year 1 only
Training of trainers at the national level	Once in year 1 only

Activities	Frequency of activities
Training at the county level	Once in year 1 only
Training at sub-county levels	Once in year 1 only
Training at the health facility level	Once in year 1 only
**Initial sensitization**	
Advocacy communications and social mobilization sub-committee meeting for planning and coordination of sensitization activities	Each month in the introduction year
National stakeholder sensitization/engagement meeting	Once in year 1 only
Stakeholder sensitization at county level	Once in year 1 only
County spokesperson and professional group sensitization	Once in year 1 only
Sub-county community dialogue day meeting support	Once in year 1 only
Community stakeholder engagement	Once in year 1 only
Community engagement for religious leaders	Once in year 1 only
**Communication / demand creation**	
Design, development, production and printing of communication materials	Once in year 1 only
Develop radio/TV message/ scripts	Once each year
Broadcasting of TV/radio messages	Once each year
Launch event at national level	Once in year 1 only
Launch event at county level	Once in year 1 only
Community mobilization through public address system (PAS)	Once in year 1 only
Routine social mobilization activities support at health facility level	Each year, shared cost
**Monitoring and evaluation**	
Monitoring and evaluation sub-committee meeting for introduction	Each year
Review, adaptation, testing of monitoring and documentation tools	
Printing and distribution of monitoring and evaluation tools	
Initial data review meeting by national, county, and sub-county officers	
**Supervision**	
Supportive supervision tool revision workshop	Once in year 1 only
Implementation (introduction and post introduction) supervision by national, county, and sub-county officers	Once in year 1 only
Routine quarterly supportive supervision by national, county, and sub-county officers	Each year, shared cost

Activities	Frequency of activities
**Service delivery**	
Vaccination administration through routine EPI or ANC clinic (fixed strategy)	Each year, shared cost
Vaccination administration through routine outreach	Each year, shared cost
**Cold chain procurement**	
Cold room walk in (capacity: 10 cu m) added at national level	In introduction year only
Cold room walk in (capacity: 10 cu m) added to all regional vaccine stores	In introduction year only
One refrigerator and two cold boxes added to all sub-county stores	In introduction year only
One cold box added to each health facility	In introduction year only

Appendix Table 2: List of areas and facilities surveyed for costing

	Name of the Institution/facility			
National level	National Vaccines and Immunization Program (NVIP)			
	National Maternal and Child Health Program			
	National vaccine store			
Regional level	Regional vaccine stores at Kisumu and Nakuru			
County level	County Health Management Team at Kilifi, Siaya, Nakuru, Marsabit, and Kirinyaga			
Sub-county level	Sub-county Health Management Team and sub-county vaccine stores at Kilifi north, Alego Usonga, Nakuru east and Saku			
Health facility level				
	County name	Sub-county name	Facility name	Facility type
	Kilifi	Kilifi North	Matsangoni Model Health Center	Health Center
	Kilifi	Kilifi North	Mtondia	Dispensary
	Kilifi	Kilifi North	Kiligi county referral hospital	Hospital
	Siaya	Alego Usonga	Siaya County Referral	Hospital

	Siaya	Alego Usonga	Ting Wang'I	Health Center
	Siaya	Alego Usonga	Mlaha	Dispensary
	Marsabit	Saku	Boru Haro	Health Center
	Marsabit	Saku	Dakabaricha	Dispensary
	Marsabit	Saku	Marsabit Referral Hospital	Hospital
	Nakuru	Nakuru East	Lanet Health Centre	Hospital
	Nakuru	Nakuru East	Bondeni Maternity Hospital	Hospital
	Nakuru	Nakuru East	Kiti	Dispensary
	Kirinyaga	Kirinyaga South	Kangu	Rural Dispensary
	Kirinyaga	Kirinyaga South	Murunduko	Health Center
	Kirinyaga	Kirinyaga South	Kimbimbi	Hospital

Appendix Table 3: Cost drivers of RSV maternal vaccine and mAb introduction and delivery

3.1. Cost drivers of RSV maternal vaccine introduction and delivery

Cost category	Financial cost (in USD)				Cost share (%)			
	Total cost (Annualized)	Introduction cost	Recurrent cost	Total full cost (initial investment + recurrent cost)	Total cost	Introduction cost	Recurrent cost	Total full cost (initial investment + recurrent)
Procurement	1,972,540		1,972,540	1,972,540	17.36%	0.00%	41.00%	15.98%
Distribution and storage	526,997		526,997	526,997	4.64%	0.00%	10.95%	4.27%
Program Planning and Coordination	640,586	388,504	252,082	640,586	5.64%	5.93%	5.24%	5.19%
Training	3,639,980	3,639,980		3,639,980	32.04%	55.57%	0.00%	29.49%
Initial sensitization	463,010	463,010		463,010	4.08%	7.07%	0.00%	3.75%
Demand creation	561,899	397,360	164,538	561,899	4.95%	6.07%	3.42%	4.55%
Monitoring and evaluation	360,382	321,292	39,090	360,382	3.17%	4.91%	0.81%	2.92%
Supervision	812,472	358,510	453,963	812,472	7.15%	5.47%	9.44%	6.58%
Service delivery	1,401,848		1,401,848	1,401,848	12.34%	0.00%	29.14%	11.36%
Waste management					0.00%	0.00%	0.00%	0.00%
Cold chain expansion	981,107	981,107		1,962,214	8.64%	14.98%	0.00%	15.90%
**Total**	**11,360,822**	**6,549,763**	**4,811,059**	**12,341,929**	100.00%	100.00%	100.00%	100.00%

Cost category	Economic cost (in USD)				Cost share (%)			
	Total cost (Annualized)	Introduction cost	Recurrent cost	Total full cost (initial investment + recurrent cost)	Total cost	Introduction cost	Recurrent cost	Total full cost (initial investment + recurrent)
Procurement	18,216,650		18,216,650	18,216,650	50.01%	0.00%	72.11%	50.05%
Distribution and storage	1,381,941		1,381,941	1,381,941	3.79%	0.00%	5.47%	3.80%
Program Planning and Coordination	1,638,911	1,094,625	544,287	1,546,899	4.50%	9.80%	2.15%	4.25%
Training	5,820,174	5,820,174		5,330,939	15.98%	52.12%	0.00%	14.65%
Initial sensitization	716,545	716,545		656,313	1.97%	6.42%	0.00%	1.80%
Demand creation	884,149	587,541	296,608	834,761	2.43%	5.26%	1.17%	2.29%
Monitoring and evaluation	877,222	838,131	39,090	806,770	2.41%	7.51%	0.15%	2.22%
Supervision	2,313,589	958,851	1,354,738	2,232,989	6.35%	8.59%	5.36%	6.14%
Service delivery	3,427,678		3,427,678	3,427,678	9.41%	0.00%	13.57%	9.42%
Waste management					0.00%	0.00%	0.00%	0.00%
Cold chain expansion	1,150,157	1,150,157		1,962,214	3.16%	10.30%	0.00%	5.39%
**Total**	**36,427,015**	**11,166,023**	**25,260,993**	**36,397,153**	100.00%	100.00%	100.00%	100.00%

3.2. Cost drivers of RSV mAb introduction and delivery

Cost category	Financial cost (in USD)				Cost share (%)			
	Total cost (Annualized)	Introduction cost	Recurrent cost	Total full cost (initial investment + recurrent cost)	Total cost	Introduction cost	Recurrent cost	Total full cost (initial investment + recurrent)
Procurement	2,604,451		2,604,451	2,604,451	20.20%	0.00%	41.04%	18.77%
Distribution and storage	526,997		526,997	526,997	4.09%	0.00%	8.30%	3.80%
Program Planning and Coordination	640,586	388,504	252,082	640,586	4.97%	5.93%	3.97%	4.62%
Training	3,639,980	3,639,980		3,639,980	28.23%	55.57%	0.00%	26.23%
Initial sensitization	463,010	463,010		463,010	3.59%	7.07%	0.00%	3.34%
Demand creation	561,899	397,360	164,538	561,899	4.36%	6.07%	2.59%	4.05%
Monitoring and evaluation	360,382	321,292	39,090	360,382	2.79%	4.91%	0.62%	2.60%
Supervision	812,472	358,510	453,963	812,472	6.30%	5.47%	7.15%	5.85%
Service delivery	2,304,770		2,304,770	2,304,770	17.87%	0.00%	36.32%	16.61%
Waste management					0.00%	0.00%	0.00%	0.00%
Cold chain expansion	981,107	981,107		1,962,214	7.61%	14.98%	0.00%	14.14%
**Total**	**12,895,654**	**6,549,763**	**6,345,892**	**13,876,761**	100.00%	100.00%	100.00%	100.00%

Cost category	Economic cost (in USD)				Cost share (%)			
	Total cost (Annualized)	Introduction cost	Recurrent cost	Total full cost (initial investment + recurrent cost)	Total cost	Introduction cost	Recurrent cost	Total full cost (initial investment + recurrent)
Procurement	29,311,289		29,311,289	29,311,289	58.80%	0.00%	75.77%	58.83%
Distribution and storage	1,381,941		1,381,941	1,381,941	2.77%	0.00%	3.57%	2.77%
Program Planning and Coordination	1,638,911	1,094,625	544,287	1,546,899	3.29%	9.80%	1.41%	3.10%
Training	5,820,174	5,820,174		5,330,939	11.67%	52.12%	0.00%	10.70%
Initial sensitization	716,545	716,545		656,313	1.44%	6.42%	0.00%	1.32%
Demand creation	884,149	587,541	296,608	834,761	1.77%	5.26%	0.77%	1.68%
Monitoring and evaluation	877,222	838,131	39,090	806,770	1.76%	7.51%	0.10%	1.62%
Supervision	2,313,589	958,851	1,354,738	2,232,989	4.64%	8.59%	3.50%	4.48%
Service delivery	5,758,635		5,758,635	5,758,635	11.55%	0.00%	14.89%	11.56%
Waste management					0.00%	0.00%	0.00%	0.00%
Cold chain expansion	1,150,157	1,150,157		1,962,214	2.31%	10.30%	0.00%	3.94%
**Total**	**49,852,611**	**11,166,023**	**38,686,588**	**49,822,749**	100.00%	100.00%	100.00%	100.00%
