# Supplementary material for: Cost of delivering childhood RSV prevention interventions to the health system in Kenya: a prospective analysis
Source: BMJ Open. 2024 Nov 21;14(11):e084207. doi: 10.1136/bmjopen-2024-084207 (PMC11590859; doi:10.1136/bmjopen-2024-084207)
Supplement: Supplementary file 2 [file bmjopen-2024-084207.reviewer_comments.pdf]

PEER REVIEW HISTORY

BMJ Open publishes all reviews undertaken for accepted manuscripts. Reviewers are asked to complete a checklist review form and are provided with free text boxes to elaborate on their assessment. These free text comments are reproduced below.

ARTICLE DETAILS

Title (Provisional)

Cost of delivering childhood RSV prevention interventions to the health system in Kenya: a prospective analysis

Authors

Baral, Ranju; Otiang, Elkanah; Odiyo, Joseph; Nyawanda, Bryan O.; Nyiro, Joyce U.; Munywoki, Patrick; Jalang'o, Rose E.; Pecenka, Clint

---

VERSION 1 - REVIEW

**Reviewer**	**1**
**Name**	**Asiri, Abdullahm**
**Affiliation**	**Ministry of Health Riyadh, public health**
**Date**	**31-Jan-2024**
**COI**	**none**

Add text on limitations and move some details from the method section to the supplements. Indicate if mAb is the long-acting vs. short-acting multi-dose one. include cost-saving for both interventions (averted hospitalization and outpatient care) if data are available.

**Reviewer**	**2**
**Name**	**De Soarez, Patricia Coelho**
**Affiliation**	**Univ Sao Paulo**
**Date**	**14-Apr-2024**
**COI**	**No competing interests**

Some methodological steps were not presented with the necessary detail or were not mentioned in the text. Please, find below my suggestions for improving the quality report of the manuscript.

Abstract

Page 3:

Provide a structured summary of objectives, perspective, setting, methods (including study design and inputs), results (including base case and uncertainty analyses), and conclusions.

Introduction

Pages 4:

Include the features of the broader context, Kenya health system, that may be affected by the economic burden of this disease. Highlight the features that may affect the payer's budget (National Vaccines and Immunization Program??) or have an impact on the provider's budget.

Report the motivation and perspective of the study.

Include study's relevance for health policy or practice decisions in Kenya. Provide a study question well specified, including details of cost components, timeframe of the study, case definition of the disease, (patient) population, and the health care setting.

Methods

Study design and Methods

Pages 5-7

1) Population

Page 4-5: Describe the characteristics of the base case population (pregnant women and birth cohort for maternal vaccine and mAb).

Page 5, line 124: Include more recent literature guidelines such as Levin, A., Boonstoppel, L., Brenzel, L. et al. WHO-led consensus statement on vaccine delivery costing: process, methods, and findings. BMC Med 20, 88 (2022). https://doi.org/10.1186/s12916-022-02278-4

2) Setting and location

Page 4-5: Include a detailed description of the geographical location (Kenya) and the particular setting of health care. This description is needed to the readers assess external validity, generalizability, and transferability of study results to their own setting.

3) Study Perspective

Health economics studies can be from the perspective of a variety of bearers of the cost of healthcare costs. For instance, Healthcare system, payers, employers, government, family, and patient. Provide and explain the choice of the study perspective (health system).

4) Time horizon

Justify why this period (5 years) is appropriate.

5) Estimating resources and costs

Describe primary or secondary research methods for valuing each resource item in terms of its unit cost. Describe any adjustments made to approximate to opportunity costs.

Were data representative of the study population?

Report the dates of the estimated resource quantities and unit costs, plus the currency and year of conversion. Describe how local currency was transformed to USD 2023 units.

6) Analysis reporting

Were the main uncertainties identified?

Was a sensitivity analysis performed on: important parameter estimates (such as cold chain, training costs), key assumptions, and point estimates?

Discussion

Pages 9-10: "Our findings suggest that, in Kenya, the unit cost of introducing and delivering new maternal RSV vaccine is slightly higher ($1.74 per vaccinated women) than introducing RSV mAb ($1.56 per vaccinated child)."

Do you think these studies results are generalizable or transferable to other settings? Please, include a paragraph discussing the generalizability and/or transferability of your results based on your cost estimates.

Discuss adequately the main uncertainties identified and their implications in the study results.

Describe any role of the funder in the identification, design, conduct, and reporting of the analysis

---

VERSION 1 - AUTHOR RESPONSE

Reviewer: 1. Dr. Abdullahm Asiri, Ministry of Health Riyadh
Comments to the Author:

Add text on limitations and move some details from the method section to the supplements. Indicate if mAb is the long-acting vs. short-acting multi-dose one. include cost-saving for both interventions (averted hospitalization and outpatient care) if data are available.

<u>Author's response:</u> Thank you for your review and comments.

Add text on limitations: Two major limitations of this study are noted in the last two paragraphs of the discussion section. These include lack of data on the product cost and product characteristics, which contributed to the higher uncertainty in cost estimates. Further, the study does not consider the availability of financing to support forthcoming RSV interventions.

Additional text on limitation is now added on Page 10, lines 333-335:

"One of the limitations of this study is that the activities costed reflect the existing health system capacities and resources needed to fill the local capacity gaps, which may limit the generalizability of the findings in different settings."

Move content from methods to supplement: While we agree that the methods section is elaborate, we believe it is important to include this level of detail in the main text given a recent recommendation on reporting the costing study findings [Vaugan et al 2020]. Further, Reviewer 2 suggested we add more details to the methods section. We therefore retained the current level of detail in the manuscript.

Indicate if mAb is long-acting vs short acting, multi dose or single dose: The mAbs considered in the current analysis are long-acting and single dose. This is added to the introduction section (page 4, lines 81-82) and also included in Table 1 (dose per course). We've further clarified this in the methods section (page 5, line 121):

> "Following the recommendations of the convening, this study generated cost estimates for the following scenarios:
>
> 1. A single-dose, long-acting mAb delivered using existing immunization visits timed closely after birth; and
> 2. A single-dose maternal vaccine delivered to pregnant women during ANC contact."

Include cost-saving for both interventions (averted hospitalization and outpatient care) if data are available: The main objective of the current study is to evaluate the cost of intervention delivery to the health system—in other words, to answer how much it costs the Kenyan health system to implement RSV interventions. Evaluating the cost savings from RSV interventions is out of scope of this analysis and, therefore, not included in the manuscript. Such data were not collected as a part of this current scope. A separate study led by a sub-group of the co-authors from the current study evaluated the economic burden of RSV in Kenya. Results from this study are currently under review at a separate journal.

Reviewer: 2, Dr. Patricia Coelho De Soarez, Univ Sao Paulo
Comments to the Author:

Some methodological steps were not presented with the necessary detail or were not mentioned in the text. Please, find below my suggestions for improving the quality report of the manuscript.

Author's response: Thank you for your review and thoughtful comments. We've revised the manuscript with the feedback and trust this has improved the clarity of the manuscript. We've addressed the specific comments in detail below.

Abstract
Page 3:
Provide a structured summary of objectives, perspective, setting, methods (including study design and inputs), results (including base case and uncertainty analyses), and conclusions.

Author's response: The abstract is now restructured per journal guidelines. We've also added strengths and limitations section after the abstract (per guidelines). The revised abstract reads as follows.

"Abstract

Objectives: To evaluate the cost of delivering childhood respiratory syncytial virus (RSV) prevention interventions to the health system in Kenya.

Design: A prospective (cost projection) activity-based costing study.

Setting: Kenya, national introduction of interventions.

Participants: Not applicable.

Interventions: A single-dose RSV maternal vaccine and a single-dose, long-acting monoclonal antibody.

Primary and secondary outcome measures: Cost per eligible target population; cost per dose administered; non-commodity cost of delivery. Costs are reported in 2023 USD.

Results: RSV interventions are expected to be delivered using existing systems; maternal vaccine utilizing the antenatal care platform and the mAb delivered similar to existing birth dose vaccines. Assuming a price of $3 per dose (for both interventions) and baseline coverage rates averaging 50% for the maternal vaccine and 86% for the mAb, the estimated cost of delivering maternal vaccine was $1.74 (financial) and $6.60 (economic) per vaccinated woman, and the cost of delivering mAbs was $1.56 (financial) and $6.27 (economic) per vaccinated child. Excluding commodity cost, the cost of delivering maternal vaccine was $1.32 (financial) and $2.72 (economic) and that for mAb was $1.23 (financial) and $2.48 (economic). Cost differences between the two interventions are driven by the anticipated baseline coverage. Health worker training, service delivery, and program planning and coordination were major cost drivers.

Conclusion: This study presents the prospective cost of new RSV intervention introduction and delivery in low- and middle-income (LMIC) settings, which is largely unknown. Cost estimates incorporate anticipated health system strengthening activities needed to deliver the future RSV interventions. These cost estimates support country- and global-level decision makers evaluating implementation feasibility and intervention affordability.

Strengths and limitations of this study:

- This is one of the first studies examining potential RSV prevention intervention delivery costs in LMICs.
- In-country experts and program representatives advised and validated key model inputs and assumptions.
- This costing study builds on the current country capacity reflecting the country specific resource needs at the time of analysis.

Introduction
Pages 4:
Include the features of the broader context, Kenya health system, that may be affected by the economic burden of this disease. Highlight the features that may affect the payer's budget (National Vaccines and Immunization Program??) or have an impact on the provider's budget.

Author's response: The main objective of the current study is to evaluate the cost of intervention delivery to the health system—in other words, to answer how much it costs the Kenyan health system to implement RSV interventions. Evaluating the economic burden of RSV disease and cost savings from RSV interventions is out of scope of this analysis and, therefore, not included in the manuscript. Such data were not collected as a part of this current scope. A separate study led by a sub-group of the co-authors from the current study evaluated the economic burden of RSV in Kenya. Results from this study are currently under publication consideration in a separate journal. Cost drivers of delivering RSV interventions are specifically discussed in the results section under cost drivers and sensitivity subsection (page 8, lines 253-271) and in the discussion section (discussed in detail on pages 9-10). Evaluating the budget impact of the RSV program was out of the scope of this costing study.

Report the motivation and perspective of the study. Include study's relevance for health policy or practice decisions in Kenya.

Author's response: Relevance and motivation for the study is elaborated on in the introduction section (page 4, lines 88-96). We've further added the following sentence to emphasize the study relevance (page 4, lines 89-90):

> "...cost of delivery evidence for maternal vaccines is nearly non-existent. This study aims to fill this gap by estimating RSV intervention introduction and delivery costs to the Kenyan health system. Understanding the costs associated with delivering future RSV interventions will allow both in-country decision makers and global policy makers to better understand the cost implications of the different intervention and delivery strategies and will inform decision-making and planning around further use of the interventions. The cost of delivery estimates generated from this study can also be used to inform cost-effectiveness and to help understand the economic feasibility of implementing such interventions as they become available."

The study perspective is described within the scope and perspective subsection under the methods section (page 5, lines 125-127):

> "Neither maternal RSV vaccine nor RSV mAb are currently available or in use in Kenya. We thus took a prospective (cost projection) approach to project costs, from the health systems perspective, of introducing and delivering these interventions in Kenya."

Provide a study question well specified, including details of cost components, timeframe of the study, case definition of the disease, (patient) population, and the health care setting.

Author’s response: We’ve added a statement to clarify the research question in the introduction section (page 4, lines 89-90):

> “This study aims to fill this gap by estimating RSV intervention introduction and delivery costs to the Kenyan health system.”

Reviewer 1 suggested the methods section is too elaborate and advised that we move some details to supplementary materials. We believe that a reasonable level of detail is included in the main text. Examples of cost categories are given in section costing approach (page 5, lines 140-142). Details on all cost components and description of each specific activity costed are provided in the supplementary table (appendix table 1).

The timeframe for the cost projections study is 5-years, which is mentioned under the sub-heading “Scope and perspective,” within the methods section (page 5, lines 128-129). The timeframe in which the study data collection was undertaken is between November 2022 and March 2023, as mentioned on page 6, lines 169-170. Health care setting and target population for the interventions are further elaborated on under the sub-heading “scenarios for program implementation and costing,” within the methods section (pages 4-5).

We believe that including a case definition of the disease is not relevant for this study as it is concerned with the cost of implementing the intervention to the government/health system rather than the economic burden of disease. However, all the relevant descriptions for this article are provided in Appendix Table 1. A separate study led by a sub-group of the co-authors from the current study evaluated the economic burden of RSV in Kenya. Results from this study are currently under publication consideration at a separate journal.

Methods
Study design and Methods
Pages 5-7

1) Population
Page 4-5: Describe the characteristics of the base case population (pregnant women and birth cohort for maternal vaccine and mAb).

Author’s response: We’ve added the following text to elaborate on the base case population (page 5, lines 129-131):

> “All pregnant women and all newborns in Kenya during the study period are assumed eligible to receive a single dose of RSV maternal vaccine and a long-acting RSV mAb, respectively.”

Page 5, line 124: Include more recent literature guidelines such as Levin, A., Boonstoppel, L., Brenzel, L. et al. WHO-led consensus statement on vaccine delivery costing: process, methods, and findings. BMC Med 20, 88 (2022). https://doi.org/10.1186/s12916-022-02278-4

Author’s response: Thank you for this recommendation. We’ve added this reference as suggested (page 6, line 145).

2) Setting and location
Page 4-5: Include a detailed description of the geographical location (Kenya) and the particular setting of health care. This description is needed to the readers assess external validity, generalizability, and transferability of study results to their own setting.

Author's response: Thanks for the comment. Since the study is not focused on a specific geography in Kenya, we did not originally include the details on the general health system in Kenya. The cost projections assume a national introduction in Kenya. We've added a sentence to clarify the current health system arrangement for vaccinating pregnant women and newborn children (page 5, lines 113-115):

> "In Kenya, most women receive vaccines at Expanded Programme on Immunization (EPI) clinics, following referral from the ANC visits. Children (including newborns) are vaccinated at EPI clinics. "

Given the activity-based costing approach of the study, generalizability of the findings from this study outside of Kenya may be limited. We've discussed this in detail in the response below.

3) Study Perspective
Health economics studies can be from the perspective of a variety of bearers of the cost of healthcare costs. For instance, Healthcare system, payers, employers, government, family, and patient. Provide and explain the choice of the study perspective (health system).

Author's response: The perspective of costing is mentioned under the sub-heading "scope and perspective" within the methods section (page 5, lines 125-127):

> "We thus took a prospective (cost projection) approach to project costs, from the health systems perspective, of introducing and delivering these interventions in Kenya. "

The choice of the health systems perspective for this study is because vaccination is primarily the function of government. We specifically purport to understand how much it costs the Kenyan government to introduce and roll our RSV prevention strategies once such products.

We've added a sentence to further clarify the perspective choice (page 5 lines 127-128):

> "The health systems perspective was chosen because vaccination is primarily the function of the Kenya Ministry of Health."

4) Time horizon
Justify why this period (5 years) is appropriate.

Author's response: The study period was based on accepted norms in the literature. See, for example, Pecenka et al 2017, Baral et al 2021, and Diawara et al 2023.

Most of the new vaccine introduction activities can be assumed to have a useful life of 3-5 years for the initial set up of related activities. With this assumption, the five year timeframe used in this analysis captures the full life of most introduction activities, generating more robust unit cost estimates.

5) Estimating resources and costs

Describe primary or secondary research methods for valuing each resource item in terms of its unit cost. Describe any adjustments made to approximate to opportunity costs.

Author's response: As detailed in the methods section, the study team conducted interviews with immunization and maternal health program representatives in Kenya to develop detailed activity maps that identified the sub-activities necessary to implement each RSV intervention (page 6, lines 150-170). We leveraged immunization program experience with recent new vaccine introductions and related financial and administrative data to inform introduction-related activities and unit costs. We also collected data from health administrative units, vaccine stores, and health facilities from five counties across Kenya to inform recurrent activities, including service delivery, and corresponding unit costs. Administrative data records from the national immunization program were used to project target population (pregnant women and birth cohort for maternal vaccine and mAb, respectively). The target population was adjusted for the anticipated coverage provided by the national immunization program.

We've added the following text to clarify the opportunity costs estimates (page 7, lines 197-200):

> "To account for the opportunity cost of staff time spent on all RSV introduction and delivery activities, we used the Kenya MOH salary scale by health worker cadre type for year 2020 based on salaries from the Salaries and Renumeration Commissions, Kenya. Time required to complete each activity was then multiplied by the average salary within a given cadre to approximate opportunity time costs."

Were data representative of the study population?

Author's response: Yes. With inputs form the MOH in Kenya, we collected data from representative samples of health administrative units, vaccine stores, and health facilities from five counties across Kenya (Kilifi, Kirinyaga, Marsabit, Nakuru, and Siaya) to capture geographic variations. The cost estimates reflect the resource requirements for the Kenyan government to implement RSV interventions.

Report the dates of the estimated resource quantities and unit costs, plus the currency and year of conversion. Describe how local currency was transformed to USD 2023 units.

Authors response: All data were collected between November 2022 and March 2023. The data collected in local currency units during the study period were subsequently validated with MOH stakeholders in 2023 and were confirmed to be reflective of 2023 prices. The local currency units were then converted to 2023 USD units using the April 2023 exchange rate from Oanda [1 USD = 134 KSH] (accessed April 25, 2023).

In the original submission, the exchange rate data source was erroneously referenced as World Bank instead of Oanda. This is corrected in the revised submission (last row Table 1).

6) Analysis reporting

Were the main uncertainties identified?

Was a sensitivity analysis performed on: important parameter estimates (such as cold chain, training costs), key assumptions, and point estimates?

Authors response: A one-way sensitivity analysis was performed by varying input values for uncertain input parameters like vaccine and mAb product price, anticipated coverage rates, allocation factor for shared inputs, etc. Product price followed by coverage rate were the most sensitive input parameters influencing estimates of cost per dose administered. We also used minimum and maximum values on variables like time spent by health workers per vaccination (observed from health facility surveys) to evaluate sensitivity of cost estimates. These variables did not have notable impact on cost per dose administration estimates. The sensitivity analysis is described on page 7, lines 218-224, and the results are presented in Figure 2.

We did not perform sensitivity analysis on cold chain or training specific costs as those activities were identified to reflect actual needs based on existing capacities and anticipated needs for future RSV interventions. These are discussed as limitations in the discussion section (pages 10-11, lines 338-345).

Discussion

Pages 9-10: "Our findings suggest that, in Kenya, the unit cost of introducing and delivering new maternal RSV vaccine is slightly higher ($1.74 per vaccinated women) than introducing RSV mAb ($1.56 per vaccinated child)."
Do you think these studies results are generalizable or transferable to other settings? Please, include a paragraph discussing the generalizability and/or transferability of your results based on your cost estimates.

Authors response: One of the limitations of this study is that activities costed reflect the existing health system capacities and resources needs to fill the local capacity gaps. This may limit the generalizability of the findings in different settings. Results generated using this approach nonetheless are useful for country decision-making as it more accurately represents the country needs. Findings from this study are informative to both country- and global-level decision-makers who are evaluating the feasibility and affordability of implementing such interventions.

The discussion section is revised to address this issue (page 10, lines 333-337):

> "One of the limitations of this study is that the activities costed reflect existing health system capacities and resources needs to fill the local capacity gaps, which may limit the generalizability of the findings in different settings. Findings from this prospective costing study nonetheless generate useful insights to help assess economic feasibility and affordability of the new products in the Kenyan context."

Discuss adequately the main uncertainties identified and their implications in the study results.

Author's response: The main cost driver based on a one-way sensitivity analysis (presented in figures 1 and 2) are product price and coverage. Both of these parameters are unknown at this time given RSV products for low-and middle-income countries like Kenya are not yet available and are not in use. The discussion section describes the implication of these results (page 10, lines 338-345):

> "Lack of data on the product cost and product characteristics extends to the higher uncertainty in cost estimates, especially for total procurement costs, and the cold chain

capacity strengthening needs. Given neither product is yet available for the LMIC market, we assumed similar prices for both products which may be overly simplistic. Nevertheless, the cost exclusive of commodity costs (i.e., delivery costs) are useful for countries assessing the long-term recurrent costs associated with new vaccine product introduction and delivery. Reasonable assumptions used in the baseline analysis and the sensitivity analysis provide a range of cost estimates and can serve as a starting point for discussions."

Describe any role of the funder in the identification, design, conduct, and reporting of the analysis.

Author's response: The funders did not contribute to the design, conduct, or reporting of the analysis. This note is added to the contribution statement section.
